# Cold-water immersion alleviates intestinal damage induced by exertional heat stroke via modulation of gut microbiota in rats

**DOI:** 10.3389/frmbi.2025.1531991

**Published:** 2025-09-09

**Authors:** Lyu Xuan, Xiaojun Sun, Baozhong Wang, Feng Chen, Yuhao Yi, Handing Mao, Yuxi Wang, Guifeng Zhao, Jiaxing Wang, Yuxiang Zhang

**Affiliations:** ^1^ Department of Critical Care Medicine, The Eighth Medical Center of Chinese People’s Liberation Army (PLA) General Hospital, Beijing, China; ^2^ Department of Emergency and Internal Medicine, Heilongjiang Armed Police Corps Hospital, Harbin, Heilongjiang, China; ^3^ Department of Critical Care Medicine, Chinese People’s Liberation Army Rocket Force Characteristic Medical Center, Beijing, China; ^4^ Department of Emergency, The Sixth Medical Center of Chinese People’s Liberation Army General Hospital, Beijing, China

**Keywords:** exertional heat stroke, gut microbiota, metabolic disorder, cold-water immersion, lipopolysaccharide

## Abstract

**Objective:**

The pathogenesis of exertional heatstroke (EHS) involves substantial contributions from gut microbiota and their metabolites. In this study, we assessed whether cold water immersion (CWI) mitigates EHS-induced intestinal damage via alterations in the microbiome.

**Methods:**

An EHS model was created with 18 Wistar rats divided into three groups, that is, the EHS group comprising rats with exertional heat stroke, the CWI group with rats with heatstroke treated with cold water immersion, and the control (CTRL) group (rats with normothermia control). Pathological changes, core temperature (Tcore), and lactic acid (Lac) and endotoxin lipopolysaccharide (LPS) levels were evaluated. Fecal samples were subjected to metagenomic shotgun sequencing and liquid chromatography–mass spectrometry for microbiota and metabolomic profiling.

**Results:**

Hematoxylin and eosin staining showed that CWI treatment significantly reduced EHS-induced intestinal congestion, edema, and necrosis compared to the EHS group. The EHS group had the highest Tcore, while the CWI group had significantly lower Tcore than the EHS group. The CWI group had significantly reduced LPS and Lac levels, similar to those observed in the CTRL group. Microbiome analysis indicated that EHS disrupted gut bacteria, with an increase in the proportion of pathogens such as *Desulfovibrio fairfieldensis, Desulfamplus magnetovallimortis*, and *Desulfococcus oleovorans* (P<0.05). CWI treatment resolved these disturbances and restored the gut microbiota to a level similar to that of the CTRL group. Metagenomic analysis showed that CWI restored gut microbiota diversity (Shannon index, P<0.05), significantly reducing the proportion of pathogenic *Desulfovibrio*. Metabolomic profiling identified key metabolites, such as inosine, hypoxanthine, guanosine, and taurine (Variable importance in projection>1, P<0.05 with P-values adjusted for multiple comparisons using the Benjamini-Hochberg method, *FDR*<0.05), differentiating between the CWI and EHS groups.

**Conclusion:**

The metabolites inosine, taurine, hypoxanthine, and guanosine correlated with restored gut microbiota, reduced proportion of *Desulfovibrio*, and attenuated inflammation (lower LPS/Lac), suggesting that their dual role in mitigating intestinal damage. These findings underscore the therapeutic potential of CWI by modulating microbial-derived metabolites, highlighting its impact on the intestinal health of patients with EHS.

## 1 Introduction

Exertional heatstroke (EHS) is a life-threatening metabolic disorder characterized by thermoregulatory failure (Leyk et al., 2019). EHS leads to the breakdown of sweat glands, dysregulation of body temperature and electrolyte balances, causing severe neurological and circulatory damage. This includes delirium, seizures, convulsions, rhabdomyolysis, acute lung injury, and in extreme cases, disseminated intravascular coagulation, multiple organ dysfunction syndrome, and death (Li Z. et al., 2021). The underlying mechanisms of EHS are complex and not entirely understood, however, it is often linked with inflammatory imbalances, oxidative stress, coagulation anomalies, rhabdomyolysis, and intestinal dysfunction.

The gastrointestinal (GI) tract, a selectively permeable barrier between the GI lumen and circulating blood, is the most extensive mucosal interface in the human body, covering approximately 250–400 m^2^ (Qiang et al., 2021). Studies indicate that EHS causes significant intestinal damage, primarily through circulatory disturbances that lead to ischemia in the GI tract. This affects the viability of intestinal cells and the permeability of the cell walls during EHS episodes (Tang et al., 2021). Moreover, mounting evidence suggests that dysbiosis in the gut microbiota plays a crucial role in the pathophysiology of intestinal injuries (Liu et al., 2022). Recent research has shown that such dysbiosis compromises the integrity of the intestinal barrier, leading to gut-derived endotoxemia, systemic inflammatory responses, and injuries across multiple organs (Jing et al., 2021). Thus, intestinal damage due to altered gut microbiota is a critical pathological feature of EHS progression. Preserving intestinal homeostasis is vital for preventing the onset and advancement of EHS.

Cold-water immersion (CWI) is often used as a recovery technique after intense physical activity (Babak et al., 2021). It is recognized as the primary treatment for EHS, with a critical emphasis on the rapid initiation of CWI once Tcore exceeds 40°C (Demartini et al., 2015). As such, CWI is regarded as the leading therapeutic approach for EHS management. The method offers several benefits, including a cold-induced analgesic effect, reduction of hyperthermia and its impacts on the central nervous system, and decreased cardiovascular strain (Ihsan et al., 2016). However, it also presents risks such as the ‘afterdrop’ phenomenon, where body temperature continues to drop post-immersion, potential mortality, overcooling, hypothermia, and organ dysfunction (Douma et al., 2020). While various mechanisms have been proposed, the exact factors that contribute to EHS improvements via CWI are still not fully understood. CWI is thought to improve cardiac blood flow and reduce the hypermetabolic state of the heart, thereby yielding therapeutic outcomes (Knapik and Epstein, 2019). The gut microbiota also plays a vital role in energy metabolism, dependent on appropriate temperature and environmental conditions for growth (Li et al., 2023). Research indicates that gut commensal bacteria can interact with pathogens, regulate virulence expression, and reduce toxin production, influencing susceptibility to EHS (Armstrong et al., 2018). (Li L. et al., 2021) found that administering *Bacillus licheniformis* for a week prior to heat exposure reduced high body temperatures, improved survival rates, and reduced immune responses and organ damage, likely by maintaining gut barrier integrity and regulating the gut microbiota to promote beneficial bacteria and reduce harmful ones. Despite these findings, the effect of CWI on EHS-induced intestinal injury remains underexplored. It is therefore essential to further investigate the role of CWI in managing EHS-induced intestinal injuries. This study aims to establish an EHS rat model and explore the mechanisms by which CWI improves EHS-induced intestinal injuries through modifications in the gut microbiome.

Microbial metabolites serve as vital mediators between bacteria and their hosts (Krautkramer et al., 2021). Specific metabolites from the intestinal flora can influence the balance between pro- and anti-inflammatory mechanisms in the gut by regulating intestinal barrier function and the oxidative stress response (Ghosh et al., 2021). Metabolomics, a sophisticated technique, allows for the identification of unique molecular ‘fingerprints’ that characterize different physiological and pathological conditions. This method is used to study the pathogenesis of metabolic diseases and the action mechanisms of therapeutic drugs (Johnson et al., 2016). Alterations in intestinal metabolic profiles are key to understanding the molecular biology of intestinal injuries resulting from EHS.

We hypothesized that CWI alleviates EHS-induced intestinal damage by modulating sulfur-metabolizing bacteria and restoring anti-inflammatory metabolites. This study aimed to test this through integrated microbiome-metabolome analysis. To test this hypothesis, the purpose of this study is to evaluate the protective effects of CWI on EHS-induced intestinal injuries and to explore the potential mechanisms involving the gut microbiome. An EHS model was established, and metabolomics analysis was performed to assess changes in the metabolic profiles of gut microbiota. Additionally, a correlation analysis was conducted to explore the interactions between gut microbiota and metabolites.

## 2 Materials and methods

### 2.1 Ethical approval

All procedures involving animals were carried out in strict accordance with the regulations and laws of China. The Institutional Ethics Committee at the Eighth Medical Center of the PLA General Hospital granted approval for these animal studies, as indicated by Acceptance Number: 3092024105301321.

### 2.2 Animals

Eighteen specific pathogen-free (SPF) Wistar rats (male, aged 8–12 weeks) from Charles River were used to minimize confounding microbial exposures. Before EHS and CWI modeling, the experimental rats were placed in a thermal environmental chamber and acclimatized at a room temperature of 25±1°C and a relative humidity of 25%, using the step training method for a period of 1-week. Animal procedures followed the NIH Guide for the Care and Use of Laboratory Animals and were approved by the Institutional Ethics Committee at the Eighth Medical Center of the PLA General Hospital.

### 2.3 EHS model establishment and CWI treatment

All wistar rats were divided into 3 groups according to the random number table method, each group comprised 6 rats: the normothermia control group (CTRL), the exertional heat stroke group (EHS), and the cold-water immersion groups (CWI). All rats were fed standard chow (LabDiet 5053) ad libitum, housed individually under identical conditions at 25±1°C with 12-hour light/dark cycles to minimize confounding variables, and confirmed to be healthy through a veterinary inspection before the experiment. The CTRL group underwent a training regimen in a room set at a temperature of 25±1°C and RH of 50±10%, following the same protocol as the experimental groups. Continuous monitoring was employed to observe any changes in the rat’s consciousness or mental status. Rats that met the diagnostic criteria for EHS were promptly removed from the chamber, and heat exposure was ceased immediately. Diagnostic criteria for EHS included neurological dysfunction (e.g., unresponsiveness to stimuli >5s). We attempt to distinguish consciousness by monitoring it every 5 minutes using reflex tests (pinna and corneal reflexes), as validated in prior studies (Wang et al., 2024). The EHS groups were successfully induced under conditions maintained at 39.5±0.3°C and 55±5% relative humidity. Rats in the CWI group underwent identical EHS induction as the EHS group (39.5±0.3°C, 55±5% humidity) until neurological dysfunction (unresponsiveness to stimuli >5s) was confirmed. After EHS induction, rats were immersed in 10.0±0.5°C water for 30 min until Tcore reached <35°C, as per the US National Athletic Trainers’Association guidelines (Proulx et al., 2003; Casa et al., 2015). The rats underwent CWI for approximately 30 min until their temperatures dropped to sub-cold levels, after which they were allowed to recover at room temperature. At 120 minutes from the start of the experiment, rats under 1.2% isoflurane anesthesia (350 mg/kg) were euthanized, and samples of plasma, colon, and feces were collected.

### 2.4 Blood, fecal and organ collection

Blood samples were collected under isoflurane anesthesia by puncturing the abdominal aorta, immediately placed into 600μL microcentrifuge tubes pre-coated with Ethylene diamine tetraacetic acid (EDTA) and lithium heparin, and then cooled on ice. Lac levels were measured using a GEM Premier 3000 analyzer (Instrumentation Laboratory, USA). For histological examination, colon tissues were excised, rinsed with a balanced salt solution, embedded in OCT, and sectioned into serial frozen slices. Sections with a thickness of 5 μm underwent staining using HE, and subsequent examination was performed with an OLYMPUS CKX41 light microscope from Japan. Additionally, plasma samples from each rat group underwent LPS analysis by Majorbio Bio-Pharm Technology Co., Ltd. in Shanghai, China.

### 2.5 Metagenomic analysis

Before DNA analysis, rat fecal samples were stored at -80°C. DNA extraction was performed using the DNA Library Prep Kit for Illumina (DP712, Tiangen Biochemical Technology (Beijing) Co. Ltd.), and microbial DNA was separated in a fume hood. The purity and concentration of the DNA were assessed using a NanoDrop 2000 spectrophotometer (Thermo Fisher Scientific, USA). DNA integrity was verified through 1.8% agarose gel electrophoresis. Fragmentation of DNA to approximately 350 bp was achieved with a Covaris M220 (Gene Company Ltd., China) for the preparation of paired-end libraries. These libraries were constructed with the TruSeq DNA Sample Preparation Kit (Illumina, San Diego, CA, USA) and sequenced on an Illumina NovaSeq platform at Majorbio Bio-Pharm Technology Co. Ltd. (Shanghai, China) using NovaSeq reagent kits according to the guidelines provided by the manufacturer.

Following sequencing, the paired-end reads from Illumina were refined by trimming and discarding low-quality sequences to eliminate host-derived contaminants. This refinement was executed using the Burrows-Wheeler Aligner toolkit (version 0.7.17). Contigs were then constructed using MEGAHIT (version 1.13). MetaGene (version 1.1.3; http://metagene.nig.ac.jp/metagene/metagene.html) was utilized for gene prediction and annotation, retrieving and translating open reading frames longer than 100 base pairs (bp). Genes with 95% sequence similarity were grouped using CD-HIT (version 4.8.1). To assess gene abundance, reads were aligned to representative sequences with a 95% similarity threshold using SOAPaligner (version 2.21) (Gu et al., 2013). For further quantification, feature counts and differential expression analysis were performed using DESeq2 (Varet et al., 2016). KEGG annotations were performed using diamond to search the KEGG database with an E-value cutoff of 1e^-5^ (Nguyen Thanh et al., 2014). Similarly, taxonomic annotations were done using diamond, targeting the nonredundant (N) DB with identical settings. Taxonomic classification was further refined using Kraken2 (version 2.0) and Bracken to improve the accuracy of species-level classification (Wishart et al., 2018).

### 2.6 Metabolomics analysis

Samples were thawed at 4°C before analysis. To each fecal sample weighing 25 mg, a cold methanol solution composed of methanol, acetonitrile, and water in a 2:2:1 ratio (v/v) was added, including an isotopically-labeled internal standard mixture (500 μl). This mixture was homogenized at low temperatures using a high-throughput tissue homogenizer to ensure uniform mixing. The solution underwent an extraction process on ice, repeated 3 times for 10 min each, to achieve full extraction. Subsequently, the mixture was purified by heating at 220°C for 30 min. The samples were then centrifuged at 12,000 rpm at 4°C for 15 min to isolate the desired components. The clear supernatant was carefully decanted into a vial for further analysis by liquid chromatography-mass(LC-MS), which integrates liquid chromatography with mass spectrometry to detect and measure molecules within the samples.

The analysis was conducted using a UPLC-Triple-TOF MS/MS system equipped with a BEH C18 column (100 mm × 2.1 mm, ID 1.7 μm) serving as the stationary phase. The mobile phase included water with 0.1% formic acid (component A), and a 1:1 mixture of acetonitrile and isopropanol with 0.1% formic acid (component B), with a flow rate of 0.40 ml/min and an injection volume of 20 μl. The temperature of the column was controlled at 40°C. The settings for the electrospray lonization (ESI) cap voltage were at 1.0 kv, injection voltage at 40 v, and collision voltage at 6 ev. Temperatures for the ion source and desolvation were set at 120°C and 500°C, respectively, while the carrier gas was flowed at 900 l/h. The system scanned a mass-to-charge ratio (m/z) from 50 to 1,250 with a resolution of 30,000. Progenesis QI software (version 2.5, Waters Corporation, Milford, MA, USA) processed the data, with metabolites identified and quantified through databases such as HMDB (version 5) (Smith et al., 2005) and METLIN (version 1.0) (Chong et al., 2018). For further metabolite identification and pathway enrichment analysis, the MetaboAnalyst platform was utilized. This included performing principal component analysis (PCA), orthogonal partial least squares discriminant analysis (OPLS-DA), and pathway analysis to visualize the differences between groups and identify key metabolic pathways (Tang et al., 2023). Raw LC-MS data were log-transformed and normalized using total ion count (TIC) normalization to correct for batch effects. The sequencing data were uploaded to the public National Center for Biotechnology Information (NCBI) (version 2023, http://www.ncbi.nlm.nih.gov/) database under BioProject accession no. PRJNA1116604.

### 2.7 Statistics and data analysis

The experiments were conducted with meticulous precision, repeating each sample thrice. To assess differences among groups, a variety of statistical techniques were employed. For binary group analyses, both the T-test and the nonparametric Wilcoxon Mann-Whitney test were administered. For evaluations involving 3 groups, methods such as nonparametric ANOVA and the Kruskal-Wallis H test were adopted. Following these assessments, the Tukey HSD method was applied for multiple comparisons. All statistical analyses involving multiple comparisons (e.g., metagenomic species, metabolites) utilized the Benjamini-Hochberg method to control the false discovery rate (FDR <0.05). Alpha and Beta diversity measures were determined using the Vegan package were assessed with permutation-based multivariate analysis of variance and Bray-Curtis distances, respectively. Low-abundance taxa (<0.001% relative abundance) were filtered to minimize noise. Metabolite data were log-transformed. Statistical analyses used R v4.3 with packages DESeq2, MetaboAnalystR and FDR adjustment was applied. The statistical evaluations utilized software tools like GraphPad Prism 9 for conducting T-tests, Wilcoxon Mann-Whitney tests, and ANOVAs, while other statistical computations were carried out using Perl. A significance threshold of P<0.05 was set for determining statistical significance. Correlations between metabolites, gut microflora, and various parameters were analyzed using Pearson and Spearman methods.

## 3 Results

### 3.1 Flowchart of the rat model

**Figure 1:**
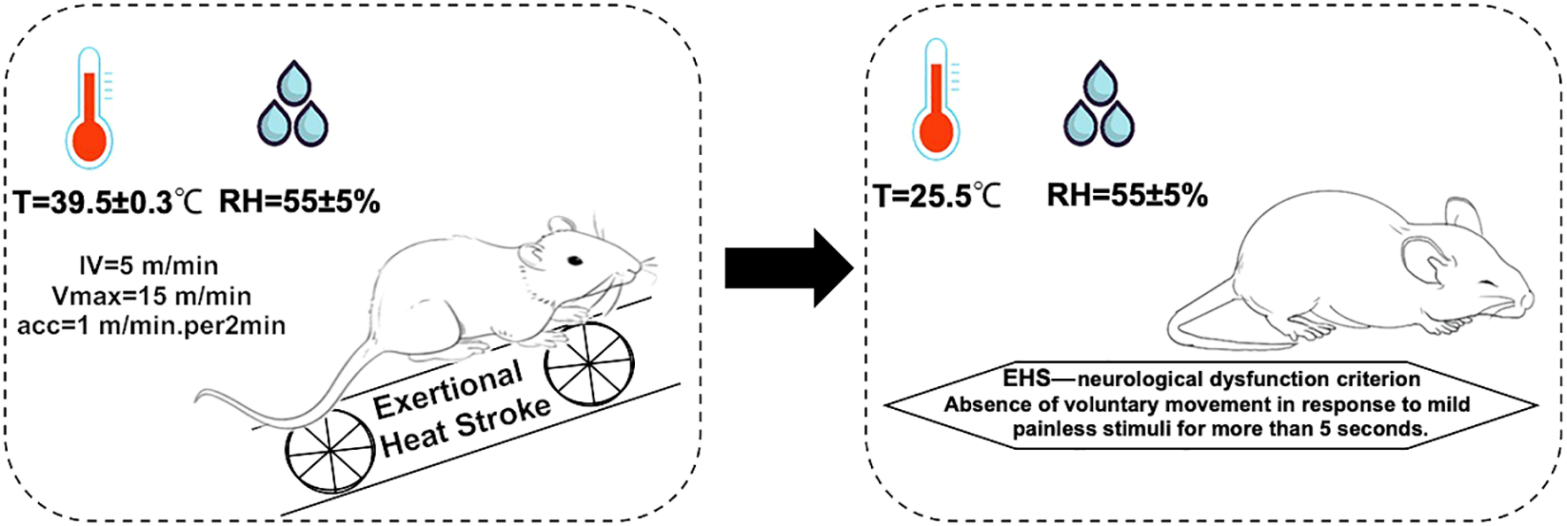
Molding regimen. The environmental conditions for the EHS model formation are as follows: the hot chamber temperature is 39.5±0.3°C, with humidity 55±5%. For fatigue training, the initial velocity is 5 m/min, increasing by 1 m/min every 2 min until reaching the maximum velocity of 15 m/min. Diagnostic criteria for EHS: failure to elicit crawling or a change in position in response to mild, painless stimuli for more than 5 s. Exertional heat stroke (EHS), Cold-water immersion (CWI), Normothermia control (CTRL).

During the EHS experiment, 6 rats showed signs of lethargy, including diminished activity, dull eyesight, slow corneal reflexes, weakened limb strength, and paleness of the mouth upon successful establishment of the model. In contrast, rats in the CWI group demonstrated a stable mental state and voluntary movements, reacted to external stimuli, maintained stable respiratory rates, although their limb grip strength was slightly reduced, and exhibited redness in the mouth and lips (**Figure 1**).

**Figure 2:**
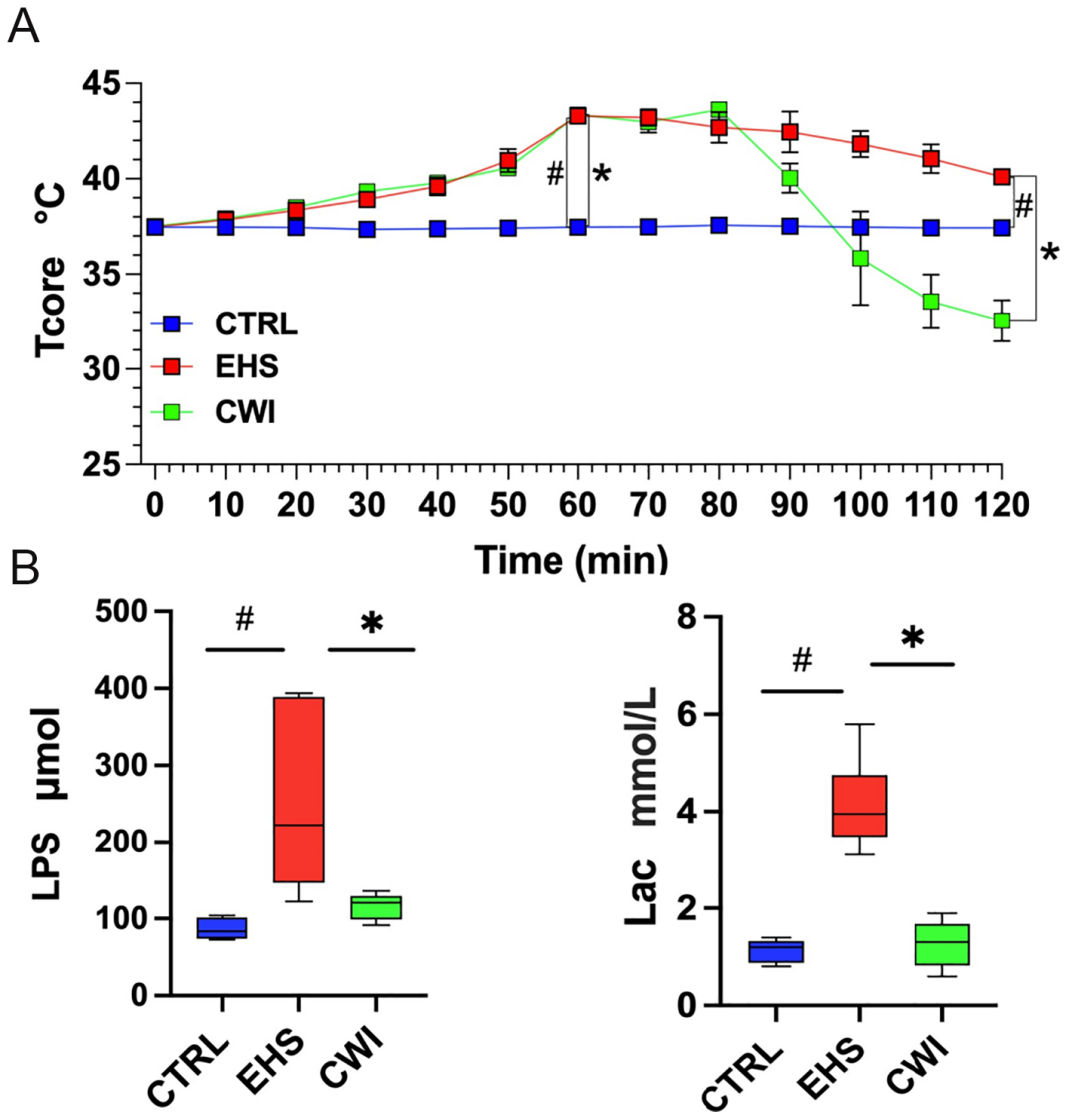
The levels of Tcore, LPS, Lac among CTRL, EHS, and CWI groups. **(A)** The Tcore of rats in each group. Blue lines denote CTRL group, red lines denote EHS group and green lines denote CWI group. **(B)** Circulatory changes of (Lipopolysaccharide, LPS), (Blood lactic acid, Lac) in each group of rats (n=6/group). ^#^P<0.05 vs. CTRL group, ^*^P<0.05 vs. EHS group; Wilcoxon Mann-Whitney ANOVA. Exertional heat stroke (EHS), Cold-water immersion (CWI), Normothermia control (CTRL).

To evaluate the effects of CWI on the physical parameters of rats subjected to EHS, Tcore was monitored continuously during the study period (**Figure 2A**). Initial Tcore measurements were consistent across the three groups before the experiments commenced (CTRL=37.5±0.12°C, EHS=37.5±0.09°C, CWI=37.5±0.16°C). 60-min post-exposure in the hot chamber, significant elevations in Tcore were observed in both the EHS and CWI groups compared to the CTRL group (EHS=43.3±0.30°C, CWI=43.4±0.22°C, P<0.05). At the 120 min time point, following the cooling procedure, Tcore in the EHS group remained significantly higher than in the CTRL group (EHS=40.1±0.26°C, CTRL=37.4±0.09°C, P<0.05), whereas the CWI group exhibited a considerable reduction in Tcore compared to the EHS group (CWI=32.6±0.10°C, P<0.05). This ‘afterdrop’ in Tcore was statistically significant. Additionally, notable decreases were recorded in LPS (EHS=250.4±105.85, CWI=116.3±15.55, P<0.05) and Lac (EHS=4.1±0.84, CWI=1.3±0.44, P<0.05) concentrations within the CWI group compared to the EHS group, aligning closely with the levels seen in the CTRL group (**Figure 2B**), raw data are in **Supplementary Tables S1** and **S2**.

### 3.2 CWI mitigates EHS-induced intestinal damage

**Figure 3:**
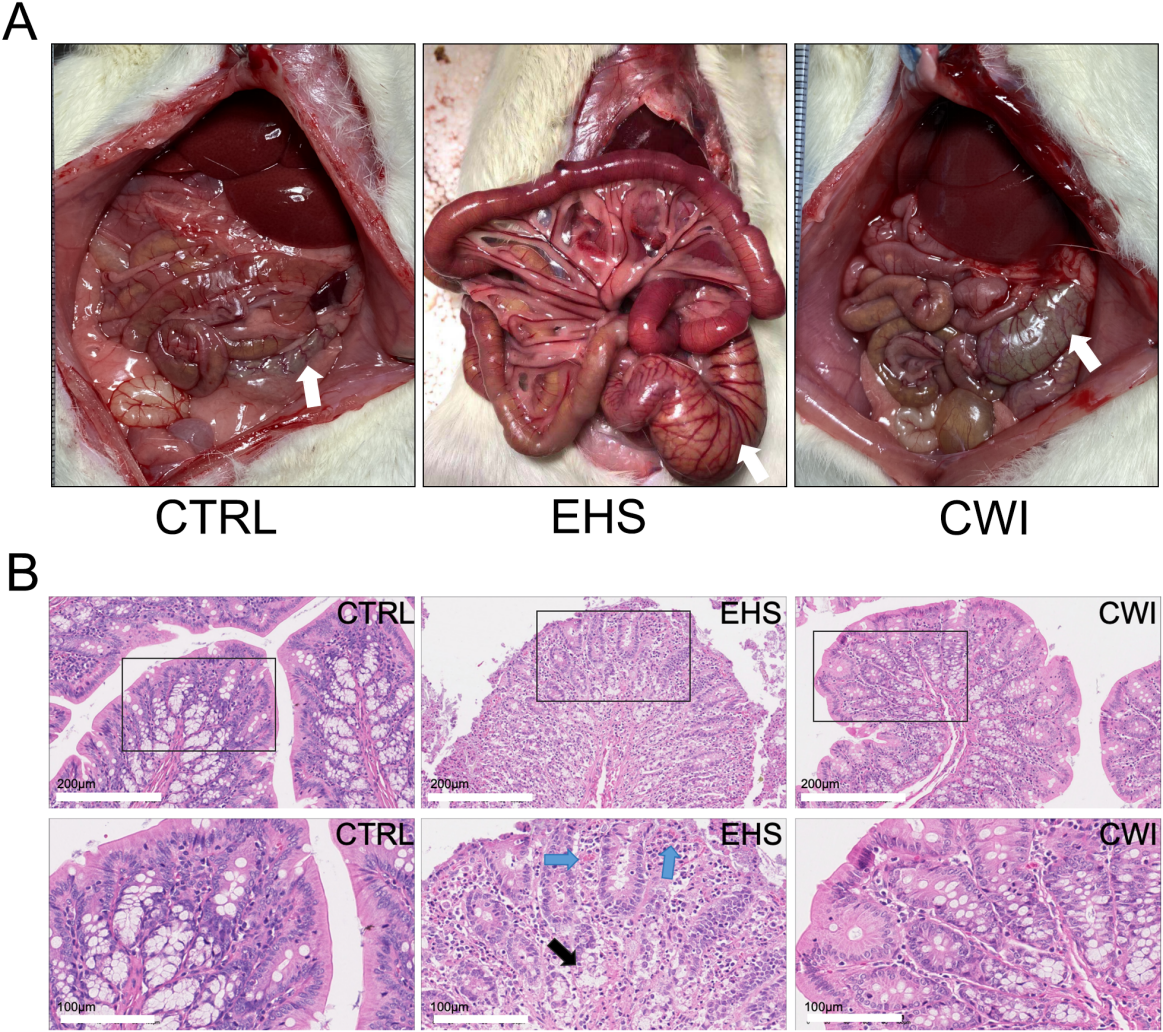
Rat anatomy and histological in each group. **(A)** The observed anatomical changes in the intestinal tract (colon tissue, white arrow) using the naked eye. **(B)** CWI mitigated EHS-induced mucosal congestion and epithelial integrity. Hematoxylin-eosin staining of the colon (200×). Black boxes represent the enlarged images below (100×). The blue arrow indicates mucosal congestion. Black arrows indicate damage to the colon lamina propria. Exertional heat stroke (EHS), Cold-water immersion (CWI), Normothermia control (CTRL).

The EHS group suffered from severe intestinal edema and bleeding compared to the CTRL group. These conditions were significantly ameliorated following CWI treatment (**Figure 3A**). Furthermore, pathological damage to the colon triggered by EHS was pronounced, but the disruption of mucosal congestion and integrity caused by EHS was effectively reduced by CWI (**Figure 3B**).

### 3.3 Intestinal microbial structure analysis

**Figure 4:**
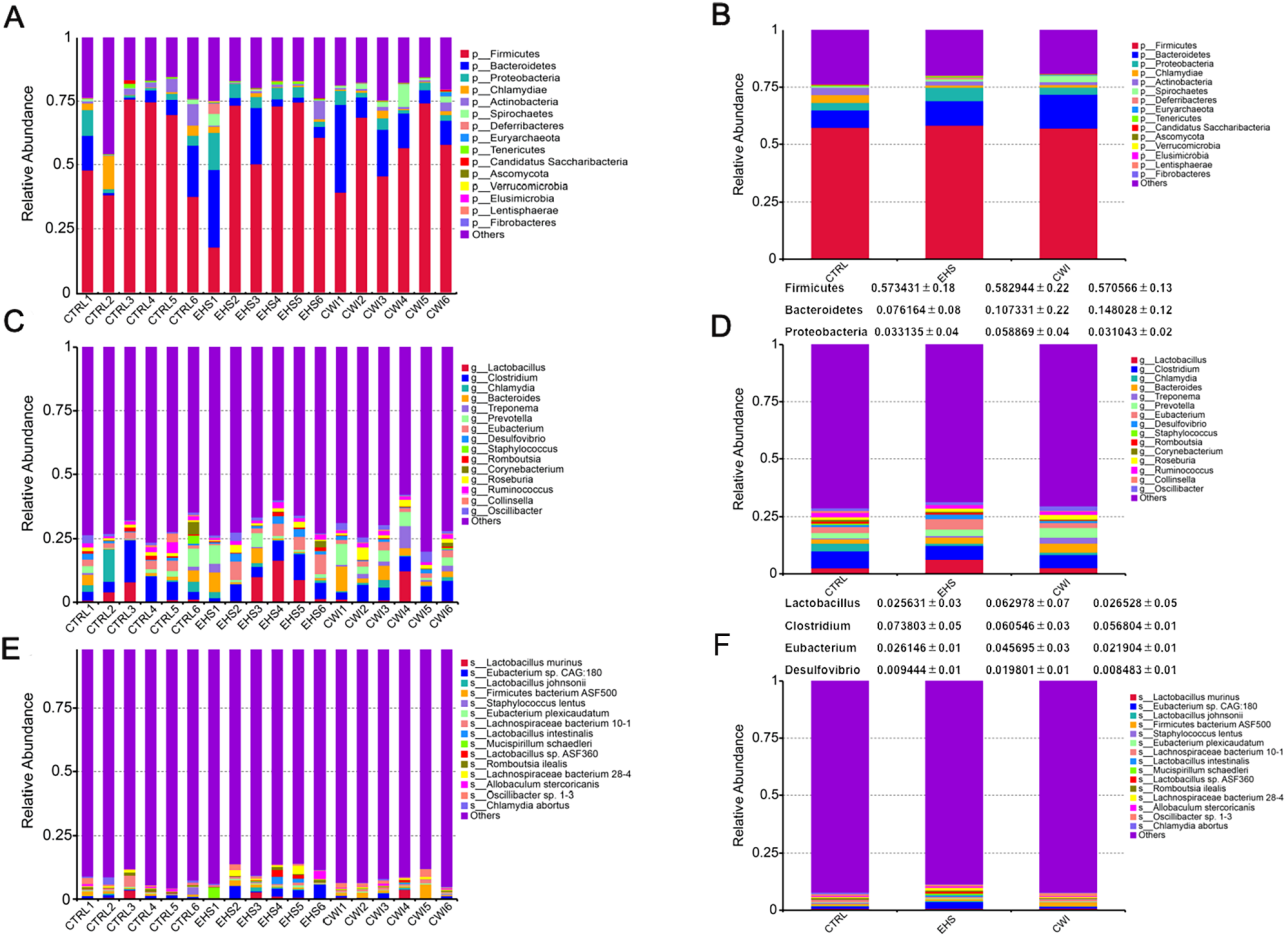
Effect of CWI on intestinal microbial diversity and structure. **(A, B)** Relative bacterial distribution at the phylum level (top 15). **(C, D)** Relative bacterial distribution at the genus level (top 15). **(E, F)** Relative bacterial distribution at the species level (top 15). Exertional heat stroke (EHS), Cold-water immersion (CWI), Normothermia control (CTRL).

To examine the influence of CWI on the prevalent species within the gut microbiota, the analysis focused on the dominant phyla and genera. As depicted in **Figures 4A, B**, the primary phyla in all study groups were Bacteroidetes, Firmicutes, and Proteobacteria, which collectively comprised over 70% of the microbiota. Although no statistical difference in the EHS group, a notable increase in the presence of Firmicutes (58.29%) and Proteobacteria (5.89%) compared to the CTRL group, which had Firmicutes (57.34%) and Proteobacteria (3.32%). Conversely, the CWI group, with Bacteroidetes (14.80%), showed a similar composition to the CTRL group (7.62%) but exhibited an increased presence of Bacteroidetes relative to the EHS group (10.73%). The research further pinpointed *Lactobacillus* (EHS 4.79% vs. CWI 2.56%), *Clostridium* (EHS 4.55% vs. CWI 4.54%) and *Eubacterium* (EHS 4.57% vs. CWI 2.19%) as the most prevalent genera within the Firmicutes, and *Desulfovibrio* as the dominant genus within Proteobacteria, as shown in **Figures 4C, D**. The proportions of *Lactobacillus murinus* and *Eubacterium* sp*14–2* were higher in the EHS group than in the CTRL group, with the average percentage of *Lactobacillus murinus* being 0.82% in the EHS group, compared to 0.79% in the CTRL group and 0.89% in the CWI group. Similarly, the average percentage of *Eubacterium plexicaudatum* was 0.41% in the EHS group, compared to 0.25% in the CTRL group and 0.30% in the CWI group, but both diminished following the CWI treatment (**Figures 4E, F**). These findings suggest that CWI may normalize the altered gut microbial composition in EHS rats by reducing the relative abundance of Proteobacteria, *Lactobacillus*, and *Eubacterium*, CWI group’s microbial profiles aligned more closely with CTRL.

### 3.4 Intestinal microbial structure analysis

**Figure 5:**
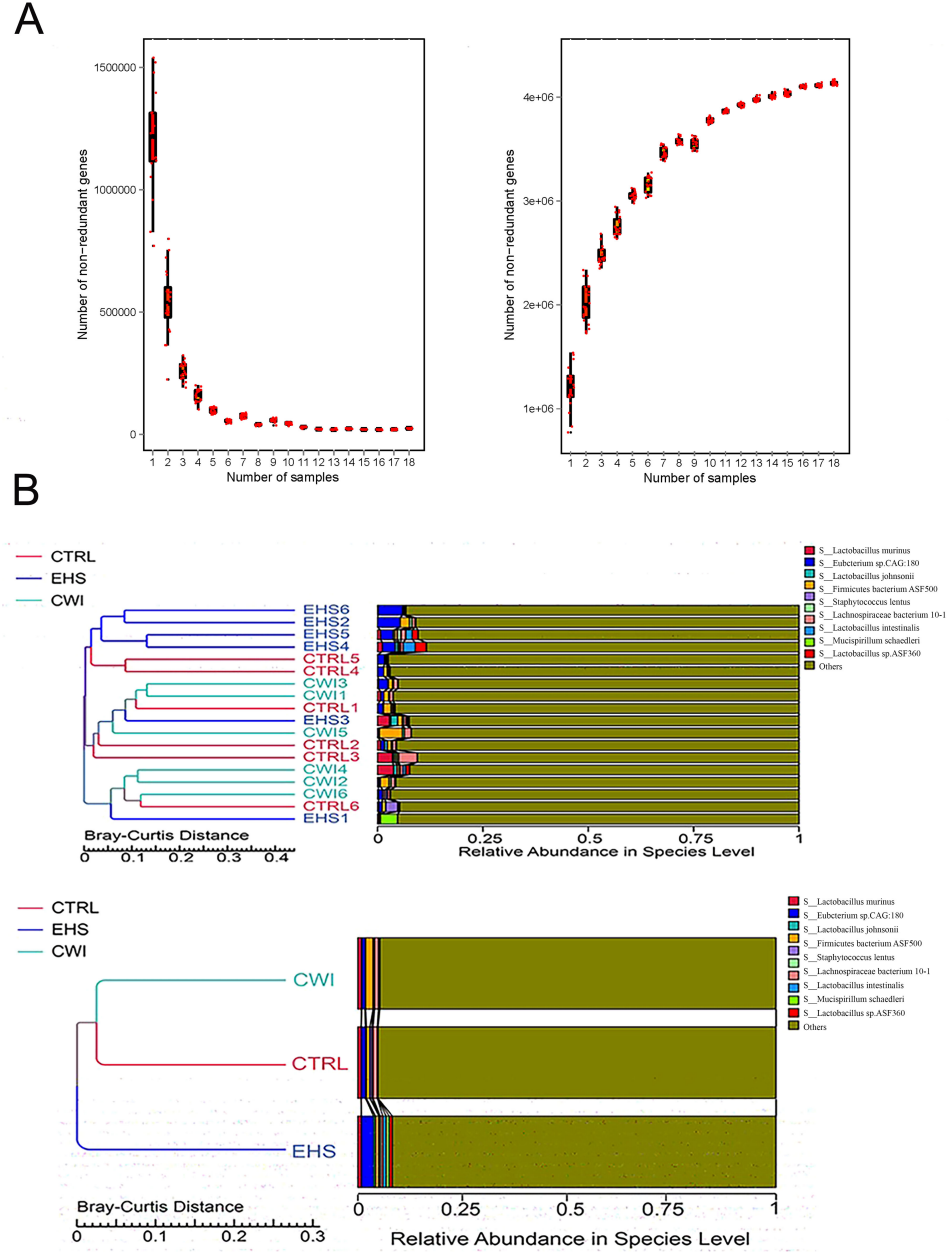
Gene counts in CTRL, CWI, and EHS groups **(A)** Sparse curves of the Core-Pan gene showing that the sample capacity is adequate. **(B)** Bray-Curtis distance-based (UPGMA) cluster tree analysis at the species level among the three groups (P<0.05 indicates significance). Exertional heat stroke (EHS), Cold-water immersion (CWI), Normothermia control (CTRL).

Gene sequencing of fecal samples was first carried out to evaluate the impact of CWI on the intestinal microbial population in rats subjected to EHS, relative to the CTRL group. The adequacy of sample sizes was verified by analyzing the core-pan gene number dilution curves (**Figure 5A**). Variations in gut microbiota were assessed using data from macrogenetic shotgun sequencing. Species-level alpha diversity was evaluated through indices such as chao1, simpson, shannon, and coverage. Following exposure to heat stroke and subsequent cold-water immersion, significant variations in fecal microbiota alpha diversity were noted. In comparison with the CTRL group, the richness indices (shannon and simpson) were significantly reduced in the EHS group, but showed marked improvements in the CWI group (P<0.05, **Table 1**). This suggests that CWI helps restore the disrupted bacterial community diversity caused by EHS. Additionally, UPGMA clustering showed that CWI restored microbial composition closer to CTRL, reversing EHS-induced dysbiosis (**Figure 5B**).

**Table 1:** Alpha diversity index of the gut microbial community at the species level for each groups.

Group	Observed_species	Shannon	Simpson	Chao1	Goods_coverage
CTRL	6713.8±821.6	8.27±0.35	0.99±0.01	6978.3±838.2	>99%
EHS	6946.8±299.5	8.1±0.24	0.98±0.01	7269±302.5	>99%
CWI	7421.2±283.6	8.6±0.36	0.99±0.01	7746.8±246.2	>99%
P value	0.061	0.0148	0.0218	0.061	0.0568

The Chao index represents the bacterial community richness. The shannon and simpson indices represent bacterial community diversity. The coverage index describes the sample sequencing coverage, community richness, evenness, and variety. Alpha diversity indices are presented as mean±S, P<0.05 indicates significance. Exertional heat stroke (EHS), Cold-water immersion (CWI), Normothermia control (CTRL).

**Figure 6:**
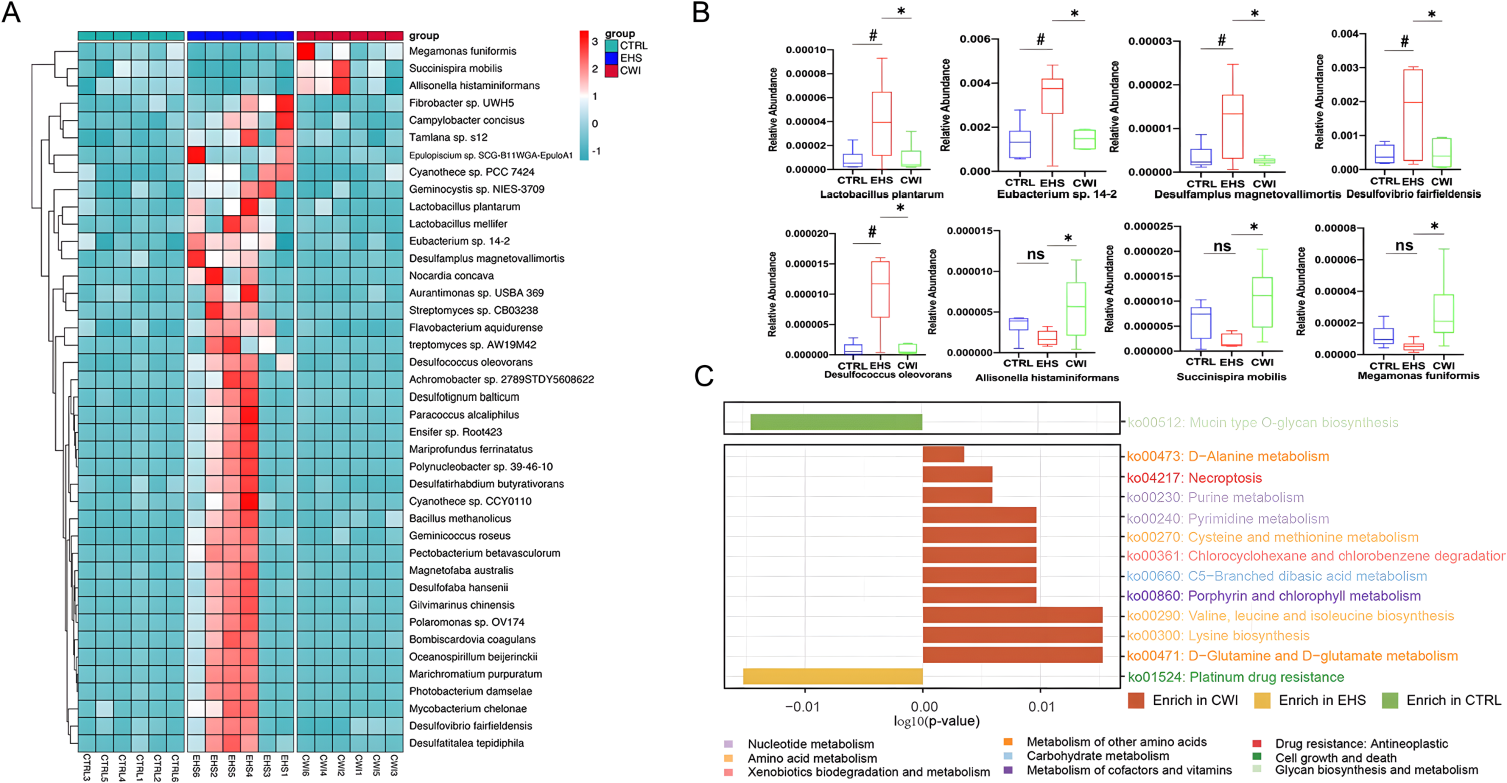
CWI regulates EHS-induced changes in microbial community and function. **(A)** Differences in enriched strains among experimental groups. **(B)** Representative differentially abundant strains in the each group. Benjamini-Hochberg method (FDR< 0.05) for balancing Type I error and statistical power in multi-omics data. **(C)** Bar graph demonstrates the difference in KEGG rectangle (KO) pathway among the three groups. The color of the KO pathway denotes the KEGG pathway class. Mann‐Whitney ANOVA. #P<0.05 vs. CTRL group, *P<0.05 vs. EHS group, ns, No significance; CTRL, Normothermia control; EHS, Exertional heat stroke; CWI, Cold-water immersion. Exertional heat stroke (EHS), Cold-water immersion (CWI), Normothermia control (CTRL).

A correlation network was established based on the relative abundance profiles of genera that were commonly abundant in both the CWI and CTRL groups (**Supplementary Figure S1A**). Further analysis revealed that within EHS group, 18 genera increased significantly (e.g., *Desulfovibrio*), while CWI reduced their abundance (P<0.05, Wilcoxon test) (Figure.S1B). The analysis also included differentially abundant species across the groups, identifying 203 species that showed differential abundance (ANOVA P<0.05), with 38 species uniquely enriched in EHS such as *Eubacterium* sp. 14-2, *Lactobacillus plantarum*, *Desulfovibrio fairfieldensis*, *Desulfamplus magnetovallimortis*, and *Desulfococcus oleovorans* (P<0.05). Notably, this elevated abundance was mitigated in the CWI group (**Figures 6A, B**). Additionally, the CWI group showed increases in the abundance of genera such as *Allisonella*, *Megamonas*, and *Succinispira* compared to the EHS group. These results highlight the positive effects of CWI in modulating the gut microbiome by reinstating microbial equilibrium in rats affected by EHS.

### 3.5 KEGG pathway analysis

Significant disparities in KEGG pathways between the EHS and CWI groups were analyzed to elucidate the functional consequences of microbial community changes following EHS and CWI interventions (**Figure 6C**). A pathway related to stress response was significantly elevated in the EHS group compared to the CWI group, while 12 pathways, predominantly associated with metabolic processes, were enhanced in both the CWI and CTRL groups (P<0.05). Among these, pathways involved in amino acid metabolism were most prevalent, particularly those related to the biosynthesis of valine, leucine, isoleucine, lysine, and the metabolism of D-glutamine and D-glutamate. In contrast, the EHS group showed enhancement in pathways linked to apoptosis, pyrimidine metabolism, and an increase in DNA repair mechanisms associated with resistance to platinum-based drugs (ko01524). These results indicate that CWI treatment can modulate microbial function, thereby providing a protective effect on the gastrointestinal tract.

### 3.6 Regulation of EHS-induced metabolic profile alterations by CWI

**Figure 7:**
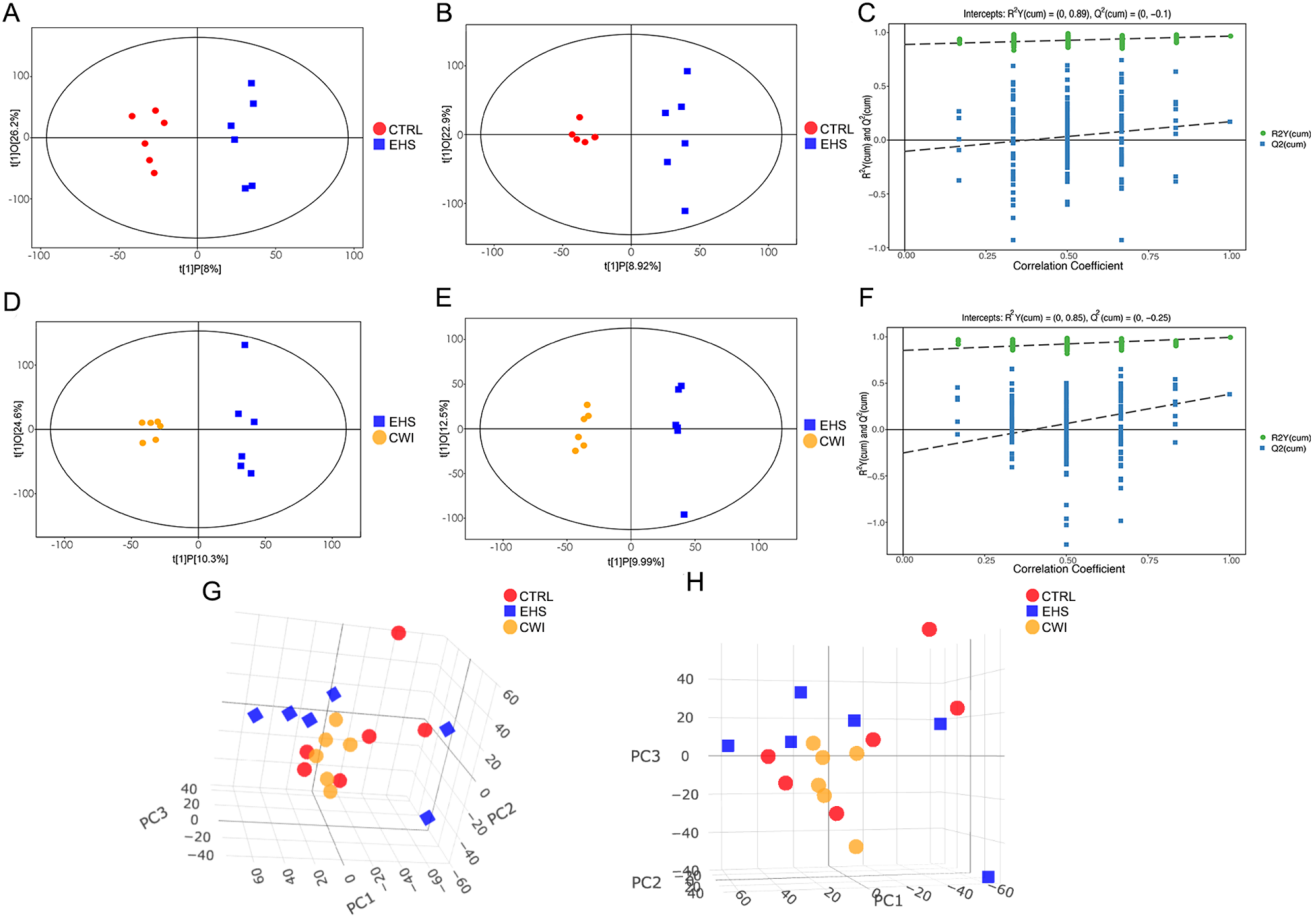
Metabolite distribution characteristics among the three groups.**(A–C)** Score scatter plot and permutation test of the orthogonal projection of potential structure discriminant analysis (OPLS-DA) model of the EHS and CTRL groups. **(D–F)** Score scatter plot and permutation test of the OPLS-DA model of the CWI and EHS groups. **(G, F)** PCA scores of experimental groups. **(A, D, G)** Positive ion mode. **(B, E, H)** Negative ion mode. Exertional heat stroke (EHS), Cold-water immersion (CWI), Normothermia control (CTRL).

To explore the distribution of metabolites across the three experimental groups and pinpoint metabolites with significant variations, PCA and OPLS-DA were utilized. The analyses highlighted clear distinctions between the EHS and CTRL groups, while the CWI group differentiated from the EHS group and aligned more closely with the CTRL group. Furthermore, an evaluation of the robustness of the OPLS-DA model was conducted, confirming the model’s effectiveness, particularly in the positive ion mode, in consistently distinguishing between the groups (**Figures 7A–H**).

### 3.7 Differential metabolite and pathway identification

**Figure 8:**
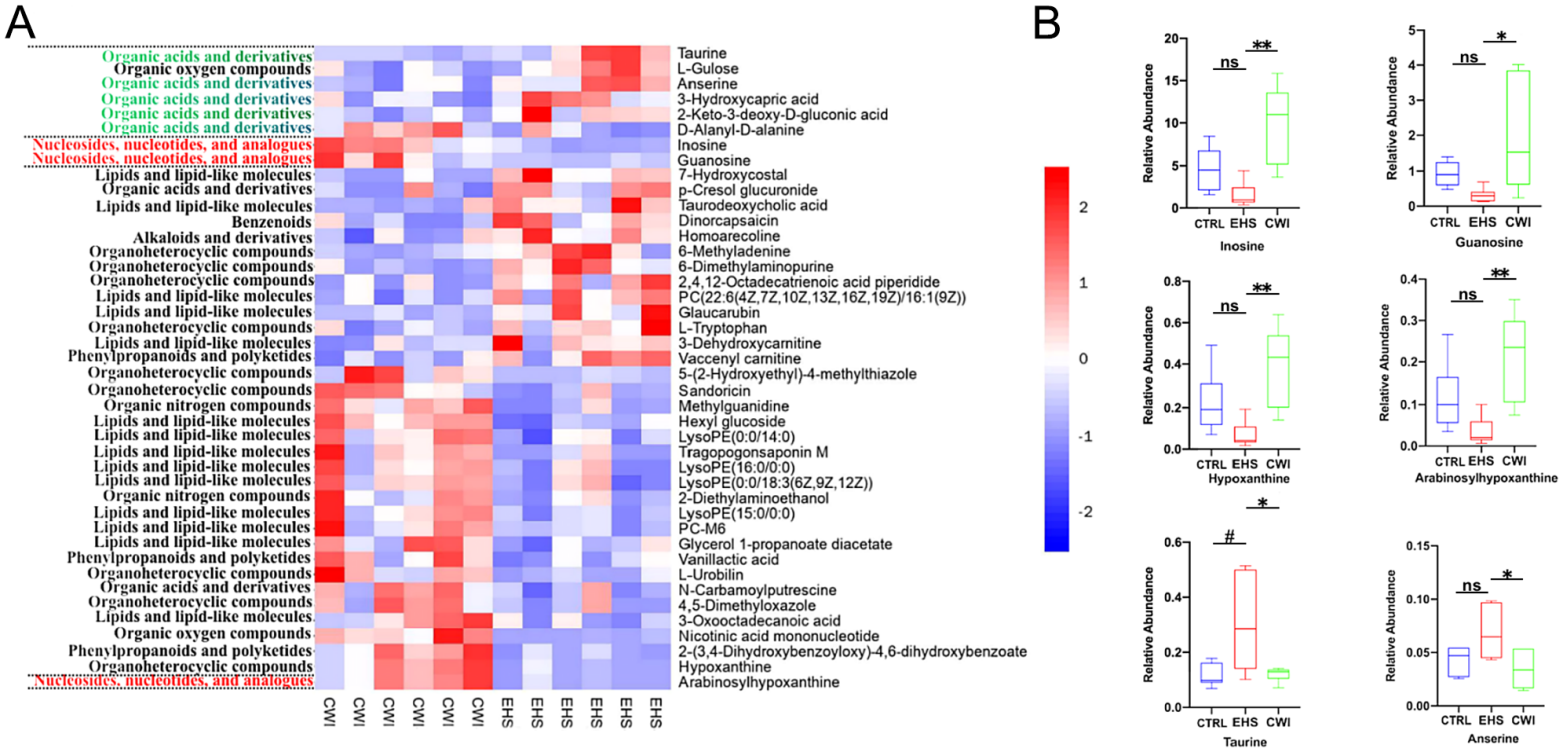
Differential metabolites between the CWI and EHS groups. **(A)** Heatmap of hierarchical clustering analysis based on metabolite z-normalized abundances. **(B)** Relative intensities of metabolites in the experimental groups. Benjamini-Hochberg method (FDR< 0.05) for balancing Type I error and statistical power in multi-omics data. ^#^P<0.05 vs. CTRL group, *P<0.05 **P<0.01 vs. EHS group. Mann‐Whitney ANOVA. Exertional heat stroke (EHS), Cold-water immersion (CWI), Normothermia control (CTRL).

Variable Importance in Projection (VIP) values from the first principal component of the OPLS-DA model were calculated to identify metabolites that significantly differ, considering VIP>1 and P<0.05 as thresholds for differential metabolites. This method facilitated the detection of key metabolites and pathways affected by CWI, revealing 42 unique metabolites including nucleotides, nucleosides, analogs, lipids, lipid-like molecules, organic acids and derivatives. Among these, notable differences between the EHS and CWI groups were observed in metabolites such as inosine, hypoxanthine, guanosine, arabinosylhypoxanthine, anserine, taurine, and D-alanyl-D-alanine (**Figure 8A**). Specifically, the concentrations of inosine (10.0±4.68 vs. 1.54±0.54, P<0.01), hypoxanthine (0.40±0.19 vs. 0.07±0.06, P<0.01), guanosine (1.98±1.58 vs. 0.31±0.20, P<0.05), and arabinosylhypoxanthine (0.22±0.10 vs. 0.03±0.03, P<0.01) were lower in the EHS group but higher in the CWI group compared to the CTRL group (**Figure 8B**). Additionally, this experiment found metabolites related to Lipid Metabolites besides the aforementioned Purine Derivatives, including LysoPE(15:0/0:0), PC-M6, LysoPE(16:0/0:0), LysoPE(0:0/18:3(6Z,9Z,12Z)), and LysoPE(0:0/14:0). Conversely, levels of taurine (0.31±0.17 vs. 0.12±0.04, P<0.05) were elevated in the EHS group but reduced in the CWI group (0.12±0.03, P<0.05), and anserine (0.07±0.02 vs. 0.03±0.02, P<0.05) also showed a decrease in the CWI group from elevated levels in the EHS group, these Amino Acids and Derivatives related metabolites also include Methylguanidine, 2-Diethylaminoethanol, and D-Alanyldalanine. Furthermore, Small Molecule Antioxidants related metabolites include Vanillactic acid, 3-Oxooctadecanoic acid, L-Urobilin, and 4,5-Dimethyloxazole, while Microbial Metabolites related metabolites include Sandoricin and Tragopogonsaponin M.

### 3.8 Association analysis of microbial metabolites

**Figure 9:**
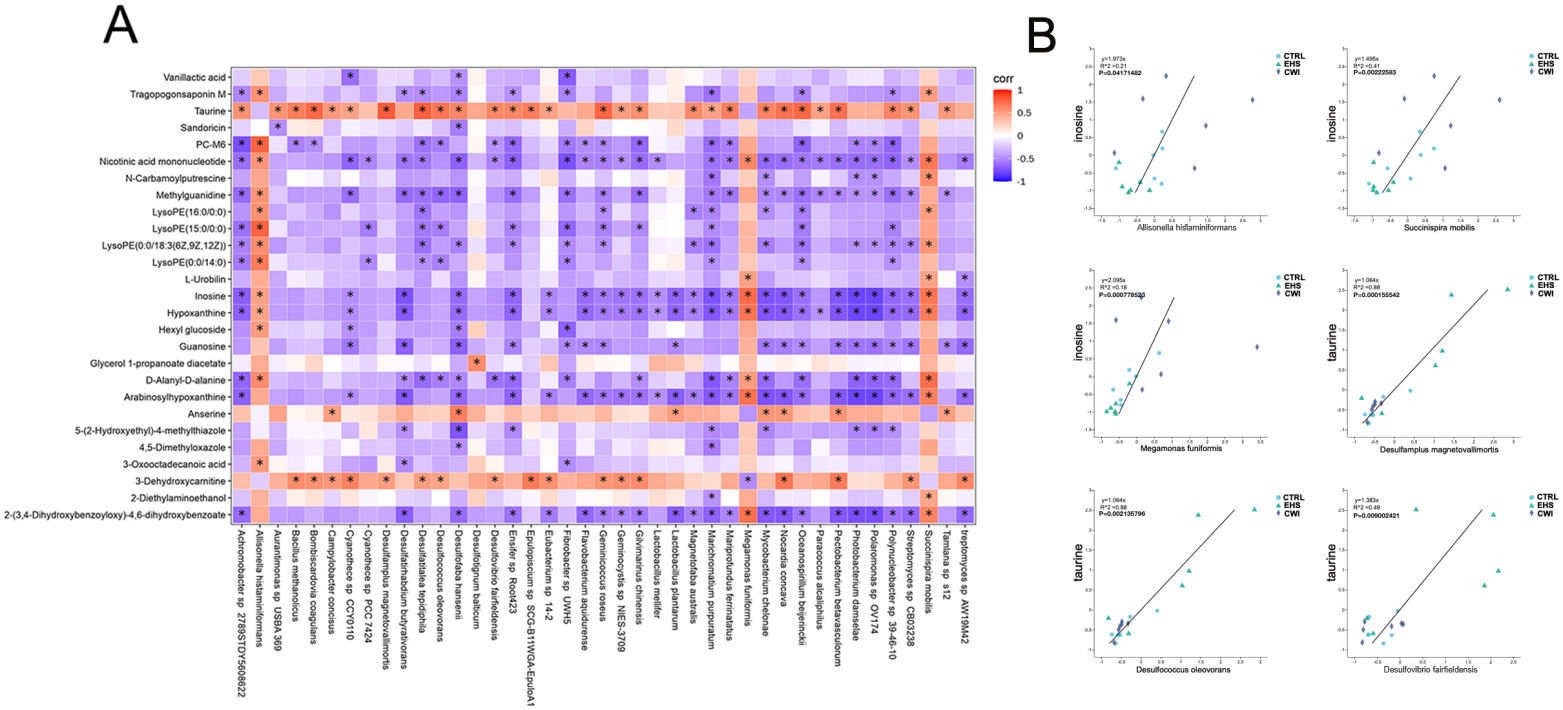
Analysis of metabolite correlation and enrichment pathways. **(A)** Correlation analysis of differential bacterial genera and metabolites between the EHS and CWI groups. **(B)** Correlation analysis of differential bacterial genera and metabolites between the EHS and CWI groups. Spearman analysis with FDR-adjusted P values (<0.05) for significance. Exertional heat stroke (EHS), Cold-water immersion (CWI), Normothermia control (CTRL).

To evaluate whether modifications in the gut microbiome serve as a mediator in the therapeutic impact of CWI, this study investigates the protective effects of CWI against intestinal damage caused by EHS (**Figure 9**). The integrated analysis of microbial genera and metabolite datasets indicated strong correlations, highlighted in **Figure 9A**. Notably, a positive link was found between taurine and its derivatives and specific species such as *Desulfovibrio fairfieldensis*, *Desulfotignum balticum*, and *Desulfococcus oleovorans* from the Proteobacteria phylum, as well as *Eubacterium* sp*14–2* from the Firmicutes phylum. Additionally, metabolites like hypoxanthine and inosine, along with their derivatives, showed a positive correlation with species such as *Allisonella histaminiformans*, *Succinispira mobilis*, and *Megamonas funiformis* from the Firmicutes phylum (**Figure 9B**). These findings suggest that changes in the gut microbiome may underlie the metabolic alterations observed in rats subjected to CWI.

## 4 Discussion

EHS ranks as the third leading cause of death among athletes participating in physical activities, with its prevalence often underappreciated (Bouchama et al., 2022). EHS typically results in severe intestinal damage, primarily due to circulatory issues that cause ischemia in the GI tract, which adversely affects the viability of intestinal cells and the permeability of cell walls during episodes of EHS (Tang et al., 2021). Recent studies suggest that dysbiosis of the gut microbiota plays a significant role in the pathophysiology of intestinal injuries.

The Dual Pathway Model (DPM) of heatstroke has identified two pathways: the ‘heat sepsis’ pathway, involves endotoxemia, systemic inflammation, and the ‘heat toxicity’ pathway, results from the thermolytic effects of heat, leading to damage in cellular structures and organs (Lim, 2018). Consequently, disturbances in intestinal flora due to heatstroke (HS) may contribute to multiple organ injuries. The established gold standard for treating athletes with EHS is to immerse the torso and lower limbs in water at 10°C, avoiding the neck and head, when Tcore exceeds 40°C and neurological symptoms are evident (Zhang et al., 2015). CWI has been shown to reduce Tcore and mitigate severe hyperemia and edema in intestinal structures, reducing damage to tight junctions in the colon and minimizing the effects of the ‘heat toxicity’ pathway (Tang et al., 2023). In models of EHS, CWI treatment has been shown to reduce severe hyperemia and edema in intestinal structures, and damage to the colon’s tight junctions was observed, thereby reducing the effects of the ‘heat toxicity’ pathway. However, the ‘heat sepsis’ pathway, which involves the intestinal flora, has yet to be fully understood.

Elevated plasma lactate levels in EHS patients suggest systemic metabolic acidosis, indicating that intestinal epithelial cells produce lactate through local anaerobic metabolism when splanchnic blood flow decreases and tissues become hypoxic (Armstrong et al., 2018). This lactic acid crosses into the cytoplasmic membrane of gram-negative bacteria, triggering the release of LPS (Armstrong et al., 2018). While CWI treatment has shown some beneficial effects, other studies have observed no changes in blood metabolite levels or lactate concentrations post-CWI (Pointon et al., 2012). While rat models effectively mimic core features of EHS, human clinical trials are essential to validate CWI protocols, such as 10–15°C immersion for 20–30 minutes, to ensure their safety and efficacy in real-world clinical settings (Comp et al., 2025). Furthermore, future research should explore the potential of combining CWI with probiotic adjuvants to enhance therapeutic outcomes, as emerging studies suggest that probiotics may support intestinal integrity and mitigate systemic inflammation associated with heat stress (Li Y. et al., 2021). Severe hyperthermia can significantly compromise enterocyte membrane integrity, which promotes the translocation of LPS into the bloodstream, triggering a systemic immune response characterized by the release of pro-inflammatory cytokines (Zuhl et al., 2014). Tight junctions play a role in this process, exhibiting increased permeability to bacterial LPS as temperatures rise, thus allowing larger molecules to pass through (Vanuytsel et al., 2014). The presence of LPS, derived from gram-negative bacteria in the mucosal lining, serves as a powerful agonist for cytokine release, exacerbating systemic inflammation in severe cases of H_2_S (Bouchama et al., 2005). A study found that levels of both pro- and anti-inflammatory cytokines were elevated following EHS, associated with exercise-induced endotoxemia due to compromised intestinal epithelial integrity (Gill et al., 2015). In models of EHS, there was a notable increase in Tcore and markers of intestinal permeability for LPS and lactate, which were effectively reversed by CWI treatment. It is hypothesized that the disruption of the intestinal microenvironment caused by EHS may exacerbate intestinal infections, leading to damage to the intestinal mucosal barrier. The metabolites produced by pathogenic bacteria could potentially disrupt the structure of the intestinal mucus layer, either directly or indirectly, leading to epithelial cell damage and accelerating the progression towards ‘heat sepsis’, ultimately resulting in damage to multiple organ functions.

Unlike prior studies focusing on probiotics (Li Y. et al., 2021), which explored the effects of probiotics on heat stroke by maintaining gut barrier integrity and modulating gut microbiota, or endotoxemia (Fuke et al., 2019), which focused on the role of gut microbiota in regulating endotoxins, we uniquely link cold-water immersion (CWI) to sulfur-metabolite regulation, highlighting its impact on the sulfur-containing metabolic bacteria in EHS rats. Sequencing results indicated that Bacteroidetes, Firmicutes, and Proteobacteria were the predominant flora, representing over 70% of the total microbial population. No differences were observed in the top 15 abundances at the phylum and genus levels among the experimental groups. However, significant variances were found in 18 genera and an additional 38 species between the EHS, CWI, and CTRL groups. It was noted that the sulfur-containing metabolic bacteria genera and strains in the EHS group were significantly disordered, and these disturbances were found to be reversible with CWI treatment. Sulfate-reducing bacteria (SRB) from the *Desulfovibrio* genus, which can be abundant in fecal matter, have been linked to ulcerative colitis. Numerous cases have associated *Desulfovibrio* with bacteremia, including sepsis, liver abscesses, acute cerebral infarction, and ulcerative colitis. Among the species, *Desulfovibrio desulfuricans* and *Desulfovibrio fairfieldensis* are frequently implicated (Singh et al., 2023). *Desulfovibrio fairfieldensis*, *Desulfotignum*, and *Desulfococcus oleovorans*, as predominant SRB, show a significant increase in abundance in the EHS groups, suggesting a potential role for SRB in exacerbating inflammation and compromising tight junction barriers in rats (**Supplementary Figure S2**). However, CWI treatment appears to restore the balance within the intestinal microecology. Additionally, alterations in small molecule metabolites within the EHS group were examined, revealing the presence of sulfur-containing taurine and indicating that CWI disrupted taurine production. Previous reports have shown a significant increase in taurine levels in patients with Inflammatory Bowel Disease (IBD) (Franzosa et al., 2019), indicating that elevated taurine concentrations may promote microbial degradation in the gut (Walker and Schmitt-Kopplin, 2021). This process leads to the production of sulfur-containing metabolites, entering the dissimilatory sulfate reduction pathway and resulting in the synthesis of H_2_S (Walker and Schmitt-Kopplin, 2021). An overproduction of H_2_S by gut bacteria has been linked to decreased mucosal integrity, attributed to reduced mucosal disulfide bonds and inhibition of colonocyte butyrate oxidation via cytochrome-c inhibition (Blachier et al., 2021). In addition, the metabolites derived from inosine, hypoxanthine, and guanosine in the CWI groups showed an increase compared to the EHS group. Inosine, a purine metabolism intermediate consisting of hypoxanthine and ribose, has been shown to suppress TNF-α in both laboratory settings and *in vivo* experiments (Lovászi et al., 2021). It also promotes the production of IL-β in macrophages activated by inflammasomes (Lovászi et al., 2021). Guanosine has been demonstrated to regulate cellular responses in T cells by reducing inflammatory cytokine production and inhibiting the NF-κB signaling pathway, thus acting as an effective anti-inflammatory agent (Luo et al., 2021). In this study, other metabolites were identified, including LysoPE(15:0/0:0), PC-M6, LysoPE(16:0/0:0), LysoPE(0:0/18:3(6Z,9Z,12Z)), and LysoPE(0:0/14:0). These compounds, which are derivatives of LysoPE and structurally similar lipid metabolites, play an important role in regulating intestinal barrier function. They enhance the stability of cell membranes, thereby mitigating intestinal barrier disruption and suppressing inflammation (Gasaly et al., 2021). Furthermore, small molecule antioxidant metabolites such as Vanillactic acid, 3-Oxooctadecanoic acid, L-Urobilin, and 4,5-Dimethyloxazole have the potential to protect the intestinal barrier by reducing oxidative stress and diminishing inflammatory responses (Gasaly et al., 2021). In addition, microbial metabolites, including Sandoricin and Tragopogonsaponin M, produced by the gut microbiota, may modulate intestinal immune responses, promote the growth of beneficial bacteria, and enhance the integrity of the intestinal barrier (Gasaly et al., 2021; Yoon et al., 2023). Consistent with these findings, there was a significant increase in the levels of inosine, hypoxanthine, and guanosine following CWI treatment in the present study, suggesting that inosine and its derivatives may act as bioactive compounds with distinct anti-inflammatory properties (**Supplementary Figure S2**). The mechanism through which CWI intervenes in the EHS model requires further study.

In this study, we identified significant perturbations in sulfur-containing microbiome-metabolites in rats from the EHS group, which were subsequently reversed following CWI treatment, restoring the microbial status to that of healthy rats. Notably, CWI treatment increased levels of inosine and its derivatives and predominantly influenced purine metabolism pathways. Interestingly, this study is the first to propose a sulfur-containing microbiome-metabolite response to disruption of intestinal tight junctions in EHS, highlighting the surprising effectiveness of CWI in reversing such intestinal damage. Although our data suggest that CWI restores microbial homeostasis, direct evidence linking specific bacteria (e.g., *Desulfovibrio*) to intestinal barrier dysfunction requires further investigation, such as fecal microbiota transplantation or gnotobiotic models.

In conclusion, we found that sulfur-microorganisms (*Desulfovibrio fairfieldensis*, *Desulfotignum*, and *Desulfococcus oleovorans*) and sulfur-containing metabolites (taurine) were significantly increased in the colons of rats in the EHS group. After CWI treatment, this upregulation was reversed, and the microbial levels were restored to levels similar to those in healthy rats. Notably, CWI treatment could lead to an increase in *in vivo* adenosine and its derivatives, and regulate the purine metabolic pathway. The results of this study provide a theoretical and experimental basis for further research on EHS-induced intestinal damage in humans and animals.

## Limitations

While our rat model replicates key characteristics of human EHS, such as hyperthermia and intestinal ischemia, interspecies differences in thermoregulatory efficiency and gut microbiota composition may influence the effectiveness of CWI. Therefore, future clinical trials are necessary to determine the optimal CWI protocols, including appropriate duration and temperature, for human application. Furthermore, this research team plans to further investigate the protective effects of CWI on intestinal injury through methods such as immunofluorescence detection of tight junction proteins and ELISA-based analysis of inflammatory cytokines.

## Data Availability

The datasets presented in this study can be found in online repositories. The names of the repository/repositories and accession number(s) can be found in the article/**Supplementary Material**.
