# Supplementary material for: Cold-water immersion alleviates intestinal damage induced by exertional heat stroke via modulation of gut microbiota in rats
Source: Front Microbiomes. 2025 Sep 9;4:1531991. doi: 10.3389/frmbi.2025.1531991 (PMC12993614; doi:10.3389/frmbi.2025.1531991)
Supplement: Supplementary file 1 [file SupplementaryFile1.docx]

**Supplemental Information**

**Cold-water immersion alleviates exertional heat stroke-induced intestinal damage by modulating gut microbiota in rats**

Lyu Xuan¹^,^², Liu Bo^1^,Xiaojun Sun², Baozhong Wang², Feng Chen³, Yuhao Yi³, Handing Mao⁴, Yuxi Wang¹, Guifeng Zhao³, Jiaxing Wang¹, Yuxiang Zhang¹

1. Department of Critical Care Medicine, the Eighth Medical Center of Chinese PLA General Hospital, No.17, Heishan Hu Road, Haidian District, Beijing, 100091, China.

2. Heilongjiang Provincial Armed Police Corps Hospital, No. 45 Wenhua Road, Nangang District, Harbin, Heilongjiang, 150010, China.

3. Department of Critical Care Medicine, PLA Rocket Force Characteristic Medical Center, No.16, Xinjiekou Outer Road, Xicheng District, Beijing 100088, China.

4. Department of Emergency, The Sixth Medical Center of Chinese PLA General Hospital, Beijing 100048, China.

**Corresponding Authors:**

Prof. Yuxiang Zhang, Dr. Jiaxing Wang, Department of Critical Care Medicine, the Eighth Medical Center of Chinese PLA General Hospital, No.17, Heishan Hu Road, Haidian District, Beijing, 100091, China.

E-mail addresses: 15810550308@163.com (Yuxiang Zhang), wangjiaxing012@163.com (Jiaxing Wang), zgfcardio@126.com (Guifeng Zhao).

**Supplemental Figure3 Description:**

Panel B displays box plots comparing the levels of LPS (left) and lactate (right) across three groups: control (CTRL), EHS, and CWI. LPS levels are significantly elevated in the EHS group compared to the CTRL and CWI groups. Lactate levels show a similar trend, with significantly higher levels in the EHS group compared to the other two groups. Statistical significance is indicated by symbols: ^#^ represents a comparison between CTRL and EHS, and * represents a comparison between EHS and CWI.

Table S1 The amount of serum LPS levels for each of the groups

| LPS/μmol  Group | Group 1 | Group 2 | Group 3 | Group 4 | Group 5 |
| --- | --- | --- | --- | --- | --- |
| CTRL | 104.1 | 77.95 | 89.4 | 72.58 | 74.31 |
| EHS | 240.2 | 154.6 | 394.1 | 204.4 | 387.2 |
| CWI | 115.2 | 91.65 | 126.3 | 101.3 | 136.2 |
| P-value | <0.05 | | | | |

Circulatory changes of (Lipopolysaccharide, LPS) in each group of rats (n=6/group). ^#^P<0.05 vs. CTRL group, ^*^P<0.05 vs. EHS group; Wilcoxon Mann-Whitney ANOVA. Exertional heat stroke (EHS), Cold-water immersion (CWI), Normothermia control (CTRL).

Table S2 The amount of serum Lac levels for each of the groups

| Lac mmol/L  Group | Group 1 | Group 2 | Group 3 | Group 4 | Group 5 |
| --- | --- | --- | --- | --- | --- |
| CTRL | 0.8 | 1.3 | 1.4 | 1.2 | 0.9 |
| EHS | 3.1 | 4 | 3.6 | 3.9 | 4.4 |
| CWI | 1.9 | 1.5 | 1.1 | 0.6 | 1.6 |
| P-value | <0.01 | | | | |

Circulatory changes of (Blood lactic acid, Lac) in each group of rats (n=6/group). ^#^P<0.05 vs. CTRL group, ^*^P<0.05 vs. EHS group; Wilcoxon Mann-Whitney ANOVA. Exertional heat stroke (EHS), Cold-water immersion (CWI), Normothermia control (CTRL).


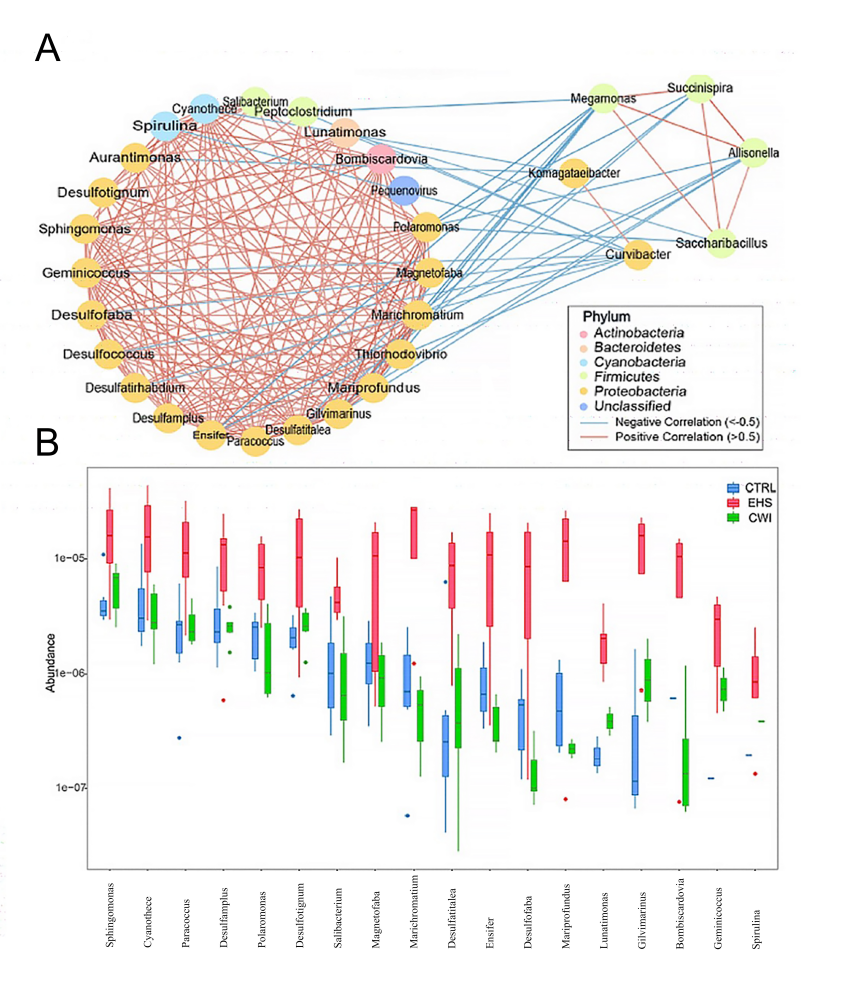


Figure. S1: Impact of CWI on gut microbial community(A, B). (A) Co-occurrence network of the differentially abundant genera between the each group (red: positive correlations; blue: negative correlations). The network shows that spearman rank correlation coefficient of >0.5 or <-0.5. Spearman analysis with FDR-adjusted P values (<0.05) for significance. (B) Box plots illustrating the differentially abundant genera shared by the each group. Mann‐Whitney ANOVA. Exertional heat stroke (EHS), Cold-water immersion (CWI), Normothermia control (CTRL).


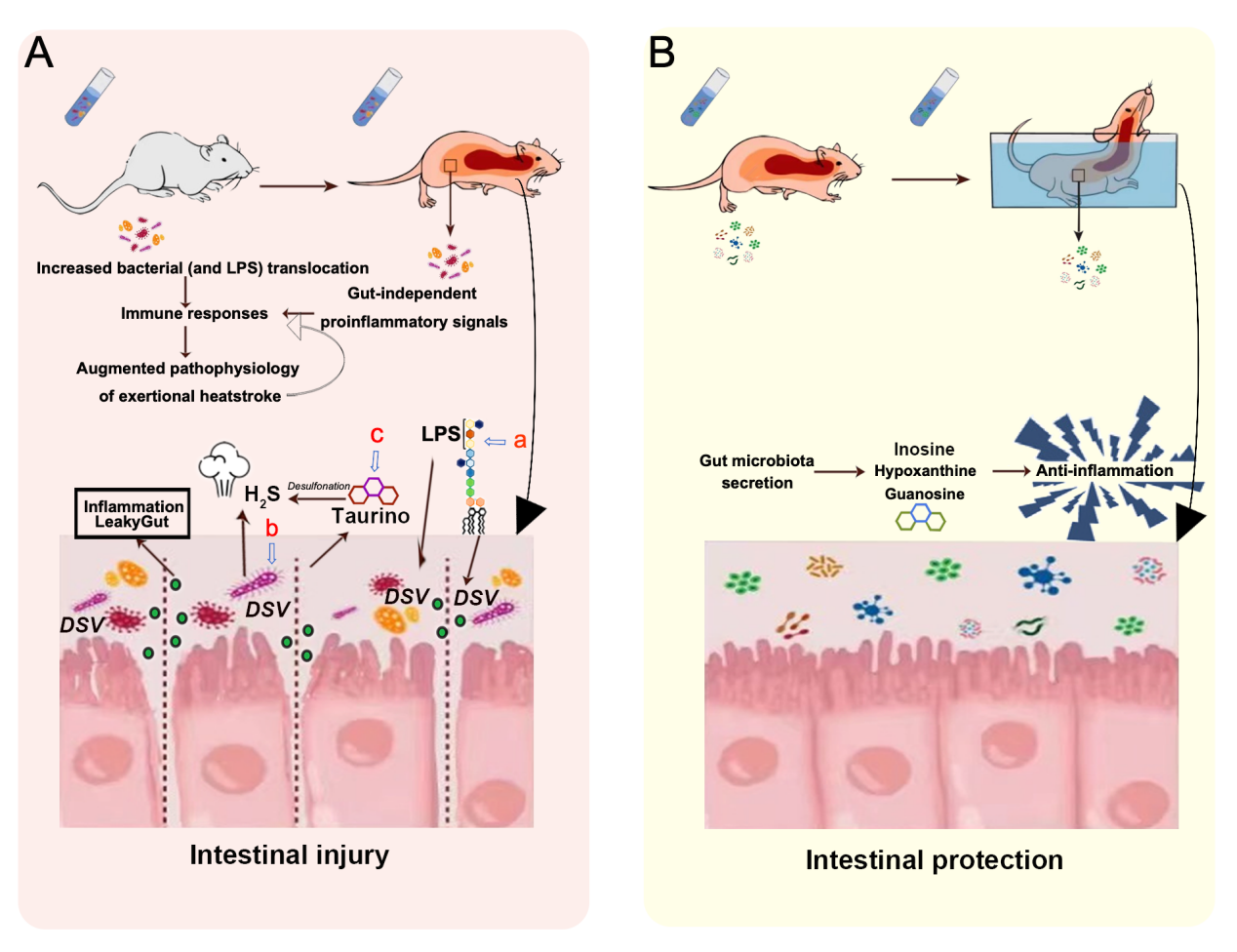


Figure S2. Schematic diagram of the mechanism of intestinal injury and intestinal protection. (A) Mechanism of intestinal injury. (B) Protective effects of CWI on the intestinal mucosa. (a: LPS levels; b: Desulfovibrio abundance; c: Taurine metabolites) Exertional heat stroke (EHS), Cold-water immersion (CWI), Normothermia control (CTRL).
